# Meteorological Table

**Published:** 1812-09

**Authors:** 


					METEOROLOGICAL TABLE,
, ' - - ?
From the 26th of July, to the 26th of August.
D. I Therm.1 I Barom.
?
?
2062
21
1022
123
24
25
26
58 55
68 55
64 54
69 57
65 58j
63 57
63 65
65 61
60 58
57 54
63 58;
64 59
57 58
58 54
59 58
63 58
61 54
70 60
71 61
68 60
69 65
73 68
81 68
74 62
74 64
68 64
67 61
70 66
69 60
72 63
70 64
29 s
296
295
29?
299
297
29?
29 *
299
30
?0*
SO2
301
30 ?
29?
29' *
30. ?
? 299
30 ?
30*
299
301
30
30
301
299
Hygrom.
Dry. Damp.
Winds. I Atmos. Variation.
9 3
- 27 5
- 17 4
1 37 17
5 16 ??]
3 ?
- 1 ?
? 3
4
27
31
17
9
3
37 15
20 9
15 17
10 ?
5 4
24 11
30 16
15 10
12 ?
2 ?
? T
7 ?
5 1
4 ?
5 6
12 15
9 ?
3 ?
2 ?
1 ?
9 ?
9:NE...
?!sw.
?.'VV.
NW..
w..
8E..
? N.
?In. ne.
ssw.
7|NE...
NW.
W..
NW.
NW.
NE..
NE.
NE.. '
N.. W,
sw?
E..
SE.S,
INE.
S..
law.,
s..
|SW..
N..
NW.
W..
W.
C.. R...
F.. R.. F..
C.. R..
C.. F ....
C. F.?
R.F.
C .F.f C..
R.F..
R... F. R... inN
R.<?...F.
C.. R...F.R.
C ... F.
C .. R.. F,
C..
C....
C...
C...
F.. ?
F...
F..
R.F..,
F ...
F....
0.. F.... R???
C . it ,. F. R...
F..
C. F...
C..F... R..F..
F.?...
C . R. F...\
Quantity of rain two inches
On the evening of the 18th, and in the night, serene lightning. On the
evening of the 19th a storm of thunder, which was very severe 8 miles to
the SW. of London.
The prevailing diseases-have not assumed any peculiar character.
Measles, scarlatina, and a very few cases of small-pox and hooping cough,
have occurred; but we have not observed many instances of diarrhoea or
pholera, in general so frequent at this season.
MONTHLY

				

